# Nutritional value of *hora* and mineral-enriched soil for cattle: Evaluation of mineral composition and physicochemical properties in southwestern Ethiopia

**DOI:** 10.1371/journal.pone.0348764

**Published:** 2026-05-14

**Authors:** Ashenafi Miresa, Taye Tolemariam, Belay Duguma, Ellen S. Dierenfeld, Geert P. J. Janssens, Abebe Nigussie, Feyissa Begna

**Affiliations:** 1 Department of Animal Science, College of Agriculture and Veterinary Medicine, Jimma University, Jimma, Oromia, Ethiopia; 2 School of Agriculture, Rural and Environmental Sciences, Nottingham Trent University, Southwell, United Kingdom; 3 Department of Nutrition, Genetics and Ethology, Faculty of Veterinary Medicine, Ghent University, Ghent, Belgium; 4 Department of Natural Resource Management, College of Agriculture and Veterinary Medicine, Jimma University, Jimma, Oromia, Ethiopia; 5 School of Veterinary Medicine, College of Agriculture and Veterinary Medicine, Jimma University, Jimma, Oromia, Ethiopia; Federal University of Santa Maria: Universidade Federal de Santa Maria, BRAZIL

## Abstract

Natural mineral water (“*hora*”) and mineral-enriched surrounding soils are potential mineral supplements for cattle, yet comprehensive data on their physicochemical properties, mineral concentration and contribution to animal nutrition have been lacking. Thus, this study aimed to evaluate the mineral composition and physicochemical properties of *hora*, and the mineral-enriched surrounding soils in the study area. *Hora* and the soil samples were collected in triplicate from each district, processed, and analyzed for physicochemical properties and mineral concentrations. The physicochemical parameters (temperature, electrical conductivity, total dissolved solids, pH, dissolved oxygen, total hardness, total turbidity, sulfate, nitrate, ammonia, chloride), and mineral concentration (calcium, potassium, sodium, magnesium, phosphorus, sulfur, iron, zinc, copper, molybdenum, selenium, and manganese in *hora* and soil samples were examined. Results revealed significant variation (p < 0.05) in physicochemical properties and mineral concentrations of *hora* across the districts. While many physicochemical properties of *hora* were found within the critical range for cattle, elevated electrical conductivity (up to 2900 *µ*S/cm), total dissolved solids (up to 1700 mg/L), and ammonia (up to 1.14 mg/L), raise concerns for its suitability for cattle. Furthermore, soil concentrations of iron (31.4 g/kg), molybdenum (200 mg/kg), and manganese (921 mg/kg) exceeded maximum tolerable limits for cattle, indicating that unsupervised soil ingestion could negatively impact animal health. While these sources offer a promising natural mineral source, their high salinity and potentially toxic mineral levels in soil necessitate careful consideration for cattle nutrition. Therefore, further research is necessary to develop sustainable management strategies for *hora* and assess its long-term implications in animal nutrition and health.

## Introduction

Natural mineral water is defined as “microbiologically wholesome” discharge from the underground sources, ensuring the absence of major contaminants, and containing a minimum of 150 mg/L of minerals [1]. These waters are a good source of essential minerals, including calcium, magnesium, potassium, sulfur, and sodium, which are important in biological reactions and physiological processes [2]. Natural mineral water sources, and the areas often associated with them, like its soils adjacent to these sources, are prevalent in both temperate and tropical ecosystems [3]. These sites frequently attract wildlife and livestock seeking mineral supplementation [4,5]. In Ethiopia, these natural mineral water sources are locally known as “*hora*” [6–8]. *Hora* water, naturally rich in various minerals often leads to the deposition of minerals in the adjacent soil due to evaporation and seepage, transforming these areas into its soil. While not a conventional natural salt lick, this soil can effectively function as a mineral supplement for animals. Animals commonly visit these areas to consume the mineral-rich soil or drink the mineral water to supplement their diet and facilitate the digestive process [9–11].

Natural mineral water and mineral-rich surrounding soils are abundantly found in different parts of the world, often linked to specific geographical features of land and geothermal activities [12,13]. In the East African landscape, natural mineral water are common and often associated with the tectonic activity of the Great Rift Valley [14]. In Ethiopia, *hora* and the adjacent its soils are found across the Afroalpine zone and the surrounding regions [6]. Local farmers and pastoralists have traditionally utilized both *hora* and its associated mineral-rich soils as sources of mineral supplements for their animals [8,15,16]. According to Kenea et al. [8], about 88.7% of farmers in these regions trek their animals to these areas for mineral supplementation. Indeed, our recent study in the same south western Ethiopian region documented these traditional practices, revealing that local farmers strongly perceive *hora* and the adjacent soil as beneficial for enhancing fattening, improving feed intake, increasing milk yield and milk fat, and boosting fertility [8]. However, prior research has also highlighted crucial concerns: despite the perceived benefits, a notable proportion of respondents reported adverse health effects, including abortions, birth defects, delayed puberty, decreased conception rates, and paralysis [8,17]. These issues were linked to excessive or unsupervised consumption of these sources. These traditional practices and reported observations underscore that *hora* and the mineral-enriched surrounding soil may be a valuable source of minerals that are otherwise difficult to obtain, but also indicate potential risks.

Minerals, including those obtained from *hora* and adjacent soil, are essential for various biological functions, and adequate intake is critical for livestock health and productivity [18,19]. Yet, as evidenced by both traditional knowledge and scientific principles, both deficiencies and excessive or imbalanced concentrations of minerals can lead to various health problems, including toxicity, reduced nutrient absorption, and impaired physiological functions [20,21]. Therefore, understanding the precise physicochemical properties and mineral profile of *hora* and the *mineral-enriched surrounding soils* is crucial for identifying their potential as sustainable and cost-effective mineral supplements, while also quantitatively assessing any potential risks associated with their unsupervised consumption in the region. This study quantifies the mineral composition and physicochemical properties of *hora* and mineral-enriched surrounding soils, building upon a previous investigation into local farmers’ perceptions and feeding practices regarding these resources in the region [8].

Despite the availability and common perceptions, *hora* and mineral-enriched surrounding soil have not been well integrated into cattle feeding systems due to a lack of detailed scientific information. While these resources are culturally well-known by local communities, their precise physicochemical properties, mineral composition, and nutritional importance in cattle feeding remains a new concept for scientific validation. Therefore, this study aims to determine the mineral composition and physicochemical properties of *hora* and mineral-enriched surrounding soil as a potential mineral supplement for cattle in the study area.

## Materials and methods

### Description of the study area

The study was conducted in the Buno Bedele zone of the Oromia Regional State, southwest Ethiopia, between December 2023 and February 2024 (dry season). The districts of Bedele, Dabo, Gechi, and Borecha were purposively selected based on *hora* availability. The average minimum and maximum temperatures of the study area range between 13–20^°^C and 24–28^°^C, respectively. The mean annual rainfall (unimodal, April to September) ranges between 1131–1500 mm. The altitudes (m.s.a.l) of the districts ranged from 1294–2374, 1268–2267, 1488–2384 and 1332–2292 for Bedele, Dabo, Gechi and Boracha district, respectively.

### Sample collection

Three prominent and actively utilized *hora* sites were purposively selected within each district, based on findings from a recent survey conducted, and further guided by documented traditional utilization patterns and perceived importance of these sites by local farmers as previously reported by Kenea et al. [8]. This resulted in a total of 12 distinct *hora* study sites (three sites per district). From each of these 12 selected *hora* sites, one composite *hora* sample and one composite soil sample (each pooled from triplicate sub-sample) were collected, totalling 24 samples (12 water; 12 soil) for laboratory analysis during the dry season (December-February).

For *hora* sampling, at each *hora* site, approximately one litre of a composite sample was collected in rinsed polyethylene plastic bottles from the surface, middle, and bottom of selected wells with an approximate one-meter depth at the sampling point (as most *hora* wells are shallow and continuously accessed by animals) in each district. The collection from surface, middle, and bottom was intended to create a representative composite sample for that specific well, effectively homogenizing the *hora* for analysis. All collected *hora* samples were immediately placed in icebox with ice packs and transported to the laboratory within 24 hours to minimize changes in physicochemical properties.

Soil samples were collected from the accessible areas immediately surrounding each well location, specifically from areas identified as mineral-enriched soil adjacent to *hora* water and animal licking activity. Approximately 500 g of soil was collected from multiple random points within each site. These sub-samples were then thoroughly pooled to form a composite sample for that site, ensuring homogeneity before analysis. After drying in the shade at ambient temperature, soil samples were transported to Horticoop Ethiopia (Horticultural) for mineral analysis.

### *Hora* sample preparation and analysis

Temperature was measured at the sampling site using a standard laboratory mercury-in-glass thermometer. Following field analyses, *hora* samples were taken to the laboratory for further mineral and physicochemical evaluations. Electrical conductivity (EC) and total dissolved solids (TDS) were measured with a conductivity meter (AD8000, Romania). The pH value was determined with a pH meter (pH-016, Canada), which was calibrated against buffer solutions of pH 4, 7, and 9.2. The dissolved oxygen (DO) was measured using a dissolved oxygen meter (CO-401, manufactured by Elmetron, Poland). The total turbidity (TT) was measured using nephelometry (CL-52D, Canada). Other *hora* parameters were analyzed according to the American Public Health Association (APHA) Standard Methods for the Examination of Water and Wastewater [22]: Total Hardness (TH) by Method 2340 C (volumetric, ethylene diamine tetraacetic acid); Ammonia (NH₃) and Nitrate (NO₃⁻) content by UV-VIS spectrophotometric methods (Section 4500-NH_3_ and Section 4500-NO_3_-, respectively); Chloride (Cl⁻) by Method 4500-Cl ⁻ B (argentometric); and Sulfate (SO₄²⁻) by Method 4500-SO₄² ⁻ E (turbidimetric).

For mineral analysis, an optimized digestion procedure was employed. A 50 ml of water sample was mixed with 4 ml of concentrated nitric acid (HNO_3_), and 1 ml of hydrochloric acid (HCl). The mixture was heated at 250^°^C for three hours until the digested volume remained at 25 ml. Then, it was filtered into a 100 ml Erlenmeyer flask and refilled up to 50 ml volume. Mineral elements were subsequently analyzed using the Inductively Coupled Plasma-Optical Emission Spectrometer (ICP-OES, Model FSH-12–2010, manufactured by Spectro Arcos, Germany). The quantified mineral elements of *hora* were calcium (Ca), potassium (K), sodium (Na), magnesium (Mg), phosphorus (P), sulfur (S), iron (Fe), zinc (Zn), copper (Cu), molybdenum (Mo), selenium (Se), and manganese (Mn).

### Soil sample preparation and analysis

Soil samples were air dried at room temperature, mechanically sieved using a 2.00 mm sieve, homogenized, and ground to fine powder to ensure homogeneity and eliminate coarse particles. Then, 1.25 g of each sample was digested with 20 mL *aqua regia* (HCl/HNO_3_ 3:1) in a beaker (open-beaker digestion) on a thermostatically controlled hot plate at approximately 95°C using a gentle heating process to facilitate digestion without boiling. The digests were heated to near dryness and cooled to ambient temperature. Specifically, 5 mL of hydrogen peroxide was added gradually to complete the digestion, and the resulting mixture was heated again to near dryness in a fume cupboard. The beaker walls were washed with 10 mL of de-ionized water, and 5 mL of HCl was added, mixed, and heated again. The resulting digest was allowed to cool and transferred into a 100 mL standard flask and brought to volume with de-ionized water.

All mineral elements, except Se were then analyzed by direct aspiration of the sample solution into an Inductively Coupled Plasma-Optical Emission Spectrometer (ICP-OES, Model FSH-12-2010, manufactured by Spectro Arcos, Germany). The ICP-OES was used to quantify the concentrations of Ca, K, Na, Mg, P, S, Fe, Zn, Cu, Mo, Mn, and Se. Soil pH and EC were measured using a pH meter (Hanna HI98130, Hanna Instruments, USA) and a conductivity meter (Eutech Instruments EC Testr 11+, Thermo Fisher Scientific, USA), respectively. Se concentration was determined using the same ICP-OES instrument, following microwave digestion and subsequent hydride generation (HG-ICP-OES) to enhance sensitivity and minimize matrix interferences. To ensure analytical robustness and sensitivity for trace element determination, the ICP-OES instrument was operated at a plasma power of 1400 W. All measurements were performed under optimized conditions, including a wavelength range of 130–770 nm and daily standardization against high-purity stock solutions (Inorganic Ventures, Germany) to correct measuring intensities. Analytical rigor was maintained through the use of reagent blanks and duplicate sample analysis, with the HG-ICP-OES method specifically validated for Se determination using internal laboratory standards. Instrumental settings were optimized to achieve high sensitivity and reproducible recovery rates consistent with standard environmental analysis protocols, ensuring that the limit of quantification (LOQ) remained within the manufacturer’s optimized performance specifications for the Arcos_SOP-ICP-OES system.

### Modelling of potential mineral intake from *hora* sources

The objective of this exercise was to model the potential daily mineral intake by adult cattle exclusively through consumption of *hora* water and associated soil. The resulting intake was used to assess the risk of exceeding MTL and to estimate the volume needed to meet daily requirements from the *hora* sources alone. This estimation models the potential mineral intake from the *hora* water and soil sources only. Daily water intake by adult cattle was estimated using the formula developed by Zanetti et al. [23] for *Bos indicus* beef cattle raised in tropical conditions:


$$\begin{array}{c} \text{WI}=\text{9}.\text{449 }+\text{0}.\text{190 }\times \text{ MBW }+\text{0}.\text{271 }\times \text{ TMAX }-\text{0}.\text{259 }\times \text{ HU }+\text{0}.\text{489 }\times \text{ DMI} \end{array}$$ (1)

where WI is water intake, MBW is the metabolic body weight calculated from an average adult cattle body weight of 250 kg (equivalent to one Tropical Livestock Unit, TLU). This resulted in an MBW of approximately 62.78 kg (250^0.75^ ≈ 62.78 kg). TMAX is the maximum temperature in degrees Celsius, which was set at a typical 26°C for the study area. HU denotes the relative humidity in percentage, with an average of 78% used for the zone. Lastly, DMI (daily dry matter intake) in kg/day was calculated as 2.5% of the cattle’s body weight, yielding an approximate 6.25 kg/day for a 250 kg animal.

Given the variability in soil ingestion by grazing cattle and the absence of specific local empirical data for the study area, a daily soil ingestion rate of 0.5 kg dry matter per head per day (0.5 kg DM/hd/day) was adopted for adult cattle. This value represents an average daily soil intake reported in existing literature for grazing cattle [24]. Moreover, our previous qualitative study [8] further substantiated the practice; local cattle exhibit distinct soil-licking behaviour at *hora* sites, providing observational evidence of consumption. This provides a pragmatic estimation of soil intake in free-ranging livestock, as direct, precise measurement of soil ingestion is logistically challenging in extensive grazing system. The estimated minerals supplied from *hora* or soils was compared against the threshold of daily mineral requirements and MTL for cattle as suggested by NRC [25].

### Statistical analysis

The analysis of *hora* and soil physicochemical properties and mineral concentration data were conducted with the help of R software (R version 4.4.1, R Foundation for Statistical Computing, Vienna, Austria) [26]. All dependent variables were tested for normality using the Shapiro-Wilk test. Differences in these parameters between districts were evaluated by one-way analysis of variance (ANOVA) with a post-hoc Tukey HSD test for multiple comparisons. The significance difference was declared at a probability level of p < 0.05.

### Ethics statement

No specific permits were required for the described field studies as the sampling sites are public traditional grazing areas. Furthermore, the sampling procedures did not involve endangered or protected species, and no animals were handled or harmed during the collection of water and soil samples.

## Results

### Physicochemical characteristics of *hora*

The physicochemical parameters of *hora* collected from various study districts are presented in Table 1. With the exception of NH_3_, Cl^-^, and TH, specific physicochemical properties showed significant (p < 0.05) variation among districts. *Hora* collected from Dabo district exhibited significantly (p < 0.01) higher temperatures and DO levels. Conversely, samples from Bedele district showed significantly (p < 0.01) higher mean EC, TDS, TT and NO_3_- concentrations. Furthermore, samples from Gechi district showed significantly (p < 0.05) higher pH and SO^4^_2_- levels.

**Table 1:** Physicochemical parameters of *hora* in the study area.

Parameters	Sample sites/Districts							Maximum permissible limit for cattle1
**Dabo**	**Bedele**	**Borecha**	**Gechi**	**Overall**	**SEM**	**p-value**
Temperature (^O^C)	24.8^a^	23.0^a^	19.0^b^	19.0^b^	21.4	0.8	0.0001	18
Electrical conductivity (μS/cm)	2552^b^	2949^a^	2257^c^	2461^b^	2555	78	<0.0001	5000
Total dissolved solids (mg/L)	1515^c^	1763^a^	1522^bc^	1540^b^	1585	31	<0.0001	3000
pH	6.6^d^	7.7^b^	7.5^c^	7.8^a^	7.4	0.1	<0.0001	8.5
Dissolved oxygen (mg/L)	3.5^a^	2.7^b^	2.3^b^	2.5^b^	2.8	0.2	0.0004	NA
Total hardness (mg/L)	520.0	521.3	522.0	521.0	521.1	0.5	0.6863	NA
Total turbidity (NTU)	2.2^c^	26.3^a^	9.4^b^	3.5^c^	10.4	2.9	<0.0001	NA
Sulfate (mg/L)	9.4^ab^	9.2^ab^	9.2^b^	9.8^a^	9.4	0.1	0.0356	1000
Nitrate (mg/L)	1.8^ab^	2.1^a^	1.7^b^	1.8^b^	1.8	0.0	0.0171	100
Ammonia (mg/L)	1.14	0.94	1.11	0.90	1.02	0.1	0.1833	NA
Chloride (mg/L)	36.2	39.8	38.6	38.4	38.2	1.3	0.8136	2000

^abc^Means across rows with different superscripts differ significantly (p < 0.05); NA = data not available; SEM = Standard error of mean; ^1^CCME [27].

### Mineral concentration of *hora*

The concentrations of macro- and micro mineral in *hora* samples are presented in Table 2, with significant variation (p < 0.05) observed across districts. For macro-minerals, *hora* collected from Bedele district showed significantly (p < 0.01) highest mean concentrations of Ca, K, and Mg, while the lowest values for these elements were recorded in Borecha and Gechi districts. Higher concentrations of Na and S were recorded from Dabo district (p < 0.01), with lower concentrations were obtained from Borecha and Gechi samples. Similarly, the mean concentration of P was significantly (p < 0.01) higher in both Dabo and Bedele districts. Except for Cu and Mo, higher concentrations of trace minerals were observed in Bedele water samples. A higher concentration of Cu was recorded from Dabo district water compared to other districts. Overall, the concentrations of all mineral elements measured were below the maximum permissible limit for cattle, although some exceeded published critical ranges (Ca, Na, Mg and S).

**Table 2:** Mineral concentrations in *hora* in the study area.

Parameters	MDL (mg/L)	Sample sites/Districts							Critical range for cattle1	Maximum permissible limit for cattle2
**Dabo**	**Bedele**	**Borecha**	**Gechi**	**Overall**	**SEM**	**p-value**
Calcium (mg/L)	<0.004	16.2^b^	48.9^a^	7.0^c^	8.2^c^	20.1	5.1	<0.0001	5-15	1000
Potassium (mg/L)	<2.177	21.9^b^	26.4^a^	5.8^c^	4.9^c^	14.8	2.9	<0.0001	2-20	NA
Sodium (mg/L)	<0.52	734.3^a^	551.4^b^	351.1^d^	382.7^c^	504.9	46.1	<0.0001	0.1-5	2000
Magnesium (mg/L)	<0.003	17.1^b^	32.4^a^	0.9^c^	1.9^c^	13.1	3.9	<0.0001	1-5	500
Phosphorus (mg/L)	<0.0001	2.4^a^	2.6^a^	1.7^b^	1.7^b^	2.1	0.1	<0.0001	1-5	15
Sulfur (mg/L)	<0.15	29.5^a^	17.3^b^	0.1^d^	2.6^c^	12.4	3.6	<0.0001	1-2	1000
Iron µg/L	<0.023	463.0^d^	1585.3^a^	1154.0^b^	884.3^c^	1021.7	123.1	<0.0001	20000-50000	50000
Zinc µg/L	<0.03	85.3^c^	131.7^a^	77.7^d^	114.7^b^	102.3	6.6	<0.0001	20000-40000	40000
Copper µg/L	0.011	51.7^a^	45.9^b^	45.0^b^	48.0^b^	47.6	0.8	0.0009	10000-20000	20000
Molybdenum µg/L	<0.051	169.7^a^	147.0^a^	145.7^a^	115.0^b^	144.3	6.4	0.0013	100-500	500
Selenium µg/L	<0.041	15.0^c^	32.0^a^	14.3^c^	24.0^b^	21.3	2.2	<0.0001	100-500	500
Manganese µg/L	<0.001	3.2^c^	53.7^a^	18.0^b^	19.0^b^	23.5	5.6	<0.0001	20000-100000	100000

^abcd^Means across rows with different superscripts differ significantly (p < 0.05); NA = data not available; SEM = Standard error of mean; MDL = Method Detection Limit; ^1^NRC (2016); ^2^CCME [27].

### Physicochemical characteristics and mineral concentrations of soil

Physicochemical properties and mineral concentrations in mineral-enriched soils surround *hora* are presented in Table 3. Soil temperatures and pH showed significant variations (p < 0.01) across the districts. Significantly (p < 0.01) higher temperatures and pH were recorded in Dabo compared to Borecha and Gechi samples. The EC of the soil samples also significantly (<0.01) varied, with the highest value recorded in the Bedele district. Among the macro-minerals, higher concentrations of Ca, Mg, and P were found in soil samples from the Bedele district (p < 0.01). Conversely, soil samples collected from the Dabo district had higher concentrations of K, Na, and S. For micro-minerals, higher concentrations of Fe, Cu and Se were found in samples from the Bedele district (p < 0.01). The highest Zn and Mo concentrations were recorded in the Bedele and Gechi districts. Although most physicochemical properties and mineral concentrations in soil were below the maximum tolerable limits for cattle, soil concentrations of Fe, Mo and Mn in the soil appear to be excessive.

**Table 3:** Physicochemical parameters and mineral concentration of the mineral-enriched soil surrounding *hora* in the study area.

Parameters	MDL (mg/kg)	Sample sites/Districts							Critical range for cattle/kg of DM1	MTL/kg of DM for cattle2
**Dabo**	**Bedele**	**Borecha**	**Gechi**	**Overall**	**SEM**	**p-value**
Temperature (^O^C)	–	21.0^a^	19.3^ab^	16.0^c^	17.3^bc^	18.4	0.6	0.0022	NA	NA
Electrical conductivity (μS/cm)	–	1514.3^b^	1616.7^a^	1455.0^c^	1541.7^b^	1531.9	17.7	<0.0001	NA	NA
pH	–	8.2^a^	8.1^b^	7.8^c^	7.9^c^	8.0	0.1	<0.0001	NA	8.5
Calcium (g/kg)	6.675	5.5^c^	21.5^a^	16.3^b^	3.2^d^	11.6	2.2	<0.0001	5-15 (g/kg)	15 g/kg
Potassium (g/kg)	2.697	3.0^a^	1.7^b^	1.4^c^	1.2^d^	1.8	0.2	<0.0001	2-20 (g/kg)	20 g/kg
Sodium (g/kg)	<0.52	3.6^a^	2.4^c^	2.7^b^	1.5^d^	2.6	0.2	<0.0001	0.1-5 (g/kg)	30 g/kg
Magnesium (g/kg)	<0.003	3.3^c^	5.8^a^	4.2^b^	1.5^d^	3.7	0.5	<0.0001	1-5 (g/kg)	40 g/kg
Phosphorus (g/kg)	4.433	1.5^a^	1.5^a^	1.5^b^	1.2^c^	1.4	0.0	<0.0001	1-5 (g/kg)	7 g/kg
Sulfur (g/kg)	<0.14	8.5^a^	5.6^b^	1.1^c^	0.2^d^	3.9	1.0	<0.0001	1-2 (g/kg)	5 g/kg
Iron (g/kg)	0.533	29.9^b^	40.5^a^	15.3^c^	39.9^a^	31.4	0.3	<0.0001	0.5-1 (g/kg)	0.5 g/kg
Zinc (mg/kg)	0.015	55.3^c^	84.5^b^	43.3^d^	90.2^a^	68.3	5.9	<0.0001	20-100 mg/kg	500 mg/kg
Copper (mg/kg)	0.059	25.7^ab^	27.2^a^	19.3^c^	24.8^b^	24.3	0.9	<0.0001	5-25 mg/kg	40 mg/kg
Molybdenum (mg/kg)	0.170	136^c^	261^a^	146^b^	257^a^	200	18	<0.0001	0.1-0.5 mg/kg	5 mg/kg
Selenium (mg/kg)	0.407	0.9^c^	1.9^a^	0.5^d^	1.2^b^	1.1	0.2	<0.0001	0.1-0.5 mg/kg	5 mg/kg
Manganese (mg/kg)	<0.007	483.2^c^	1104.3^b^	317.9^d^	1777.3^a^	920.7	173.4	<0.0001	20-100 mg/kg	500 mg/kg

^abc^Means a3cross rows with different superscripts differ significantly (p < 0.05); NA = Data not available; MTL = Maximum tolerable limit; SEM = Standard error of mean.; MDL = Method Detection Limit; ^1^NRC (2016); ^2^CCME [27].

### Estimated mineral contribution form *hora* and soil in relation to cattle requirements

The daily mineral intakes and potential contribution of *hora* and soils in relation to the daily mineral requirements of local Zebu cattle are presented in Table 4. Except for Na and Se, concentrations of macro- and trace minerals in *hora* were insufficient to meet daily mineral requirements when relying solely on *hora* consumption. Conversely, based on the estimated daily ingestion of soil, cattle could consume an adequate gross amount of minerals from soils to satisfy their daily mineral requirements.

**Table 4:** Estimated mineral contribution from *hora* and soils in relation to the mineral requirements of cattle in Ethiopia.

Minerals	Estimated Mineral intake (g day^-1^)		Gross Mineral Requirements(g day^-1^)*	Estimated Amount Needed to Meet Requirement (day^-1^)		Maximum tolerable limit (day-1)*
***Hora***	**Soil**		**Water (L)**	**Soil (g)**
Calcium	0.66	5800	9.9	202	0.9	112.5 g
Potassium	0.36	900	14.8	562	8.2	150 g
Sodium	9.91	1300	2.5	3.4	0.9	225 g
Magnesium	0.44	1850	4.9	153	1.3	45 g
Phosphorus	0.04	700	4.9	1904	3.5	52.5 g
Sulfur	0.40	1950	3.7	126	0.9	37.5 g
Iron	21.60	15700	123.7	77	3.9	3.8 g
Zinc	2.70	34000	74.3	371	1.1	3750 mg
Copper	2.70	12150	24.7	124	1.0	300 mg
Molybdenum	2.70	100000	12.4	62	0.1	150 mg
Selenium	1.35	550	0.2	2.5	0.2	37.5 mg
Manganese	1.35	460500	49.5	495	0.1	3750 mg

*Gross mineral requirements and maximum tolerable limits for adult cattle were obtained from established nutritional guidelines of NRC [25].

## Discussion

The physicochemical characteristics and mineral concentration of *hora* and mineral-enriched surrounding soil across the study districts reveal a complex interplay between nutritional benefits and potential metabolic risks. While most parameters generally fell within acceptable limits for cattle consumption, notable variations among districts and higher concentration of specific parameters warrant careful consideration. A primary concern identified in this study is the impact of water quality on palatability and subsequent intake. For instance, *hora* samples from Bedele district exhibited notably elevated levels of EC, TDS, and TT. Increased TDS in water, particularly due to high concentrations of dissolved salts like sodium, chloride, sulfates of sodium, and magnesium, can directly impact water palatability by altering its taste (often bitter or salty) and odor, potentially leading to reduced water intake by cattle [28,29]. This reduction water in intake, in turn, can negatively affect feed intake, nutrient utilization, and overall productivity [30,31]. Beyond palatability, high TDS driven by excess minerals like Ca, Mg, and P can lead to serious health issues such as urinary calculi (urolithiasis) in male ruminants due to increased urine saturation [32]. High sodium levels, often associated with high TDS, also pose a risk of salt poisoning if other water sources are limited [33].

Elevated turbidity, while not directly toxic, is a critical indicator of poor water quality [34]. High turbidity can harbor and shield waterborne pathogens (bacteria, viruses, protozoa) from natural defenses or potential disinfection, thereby increasing the risk of waterborne illnesses such as gastrointestinal distress, diarrhea, and subsequent reductions in feed intake and overall productivity [35]. Furthermore, the unappealing appearance of highly turbid water can significantly reduce its palatability, leading to decreased water consumption by cattle, which in turn compromises their hydration, nutrient utilization, and overall performance [36]. The elevated TT observed in Bedele samples might be attributed to contamination from animal waste, soil erosion, algal blooms, and human activities, as these wells are publicly open. Consistent with this results, previous studies have reported that elevated TT in open water wells is primarily caused by suspended particles from soil, organic matter, and human activities [37]. Conversely, the relatively lower TT in *hora* from Dabo samples supports the effectiveness of protective measures from contamination, as these *hora* have fenced access [8].

Beyond these physicochemical concerns, the mean temperature of *hora* (21°C) exceeded the preferred range for cattle (18°C) [27]. This elevated water temperature is not merely an aesthetic issue; it actively promotes microbial growth and negatively impacts water quality in terms of taste, odor, color, and corrosion [38], thereby potentially reducing water intake. The low DO values observed in *hora* most likely evolve from these higher temperatures, as oxygen dissolves less efficiently at increasing temperatures [39]. Furthermore, *hora* in this study is not only hypertonic but also classified as very hard water as classified according to Albertini et al. [40]. Although no specific health-based guideline exists for TH for cattle, the consumption of hard water can influence water intake, disrupt Ca and Mg balance, and reduce nutrient digestibility, as reported by Yirga et al. [41]. Despite the high mineral density relative to typical freshwater, *hora* consumption alone is insufficient to meet the total daily requirements for most macro- and trace minerals. However, its role as a targeted supplement for Na and Se is significant. The saline nature of *hora* Na offers a pragmatic solution to the Na deficiencies commonly reported in free-ranging Ethiopian cattle [42].

The analysis of the mineral-enriched soils surrounding *hora* samples revealed high to very high concentrations of minerals, presenting both potential benefits and risks. While these soils can be a beneficial source of mineral supplementation, the extreme concentrations of Fe, Mo, and Mn exceeded MTL. The high mineral concentrations in the soil are attributed to local geological factors and evaporation, which significantly concentrates dissolved minerals in surface soils [43]. Although the soils being fairly rich in Cu, the extremely high concentrations of its main antagonists Fe (extremely high), Mo and Mn (very high), S and Zn pose a significant risk of inducing secondary Cu deficiency [44]. This particularly concerning given that Cu deficiency in cattle appears widespread in south western Ethiopia [45]. Antagonistic interactions, where Fe, Mo and Zn minerals interfere with Cu absorption and metabolism [44], are highly probable given the extreme values, especially for Fe. Furthermore, the alkaline pH in the soil does not favor the absorption of most minerals, potentially exacerbating these imbalance. Conversely, in the rare case of Fe deficiency in animals in the region, a few grams of this soil could significantly contribute to daily Fe intake, assuming good bioavailability. While *hora* is a primary source of Na and Se, minerals like Fe, Mn, and Mo are overwhelmingly contributed by soil ingestion, reflecting their high concentrations in the local soil matrix.

### Limitations and future directions

It is essential to contextualize these findings within the study’s methodological constraints. This research was designed as an exploratory characterization of the *hora* resource; thus a primary limitation is the absence of a comparative baseline from sites distal to the *hora* springs. Without samples of drinking water and soil away from these specific points, the exact magnitude of enrichment relative to the basal environmental status of the study area cannot be fully quantified. Furthermore, our modeling of mineral intake excluded background forage and alternative water sources. Given that forage constitutes the largest portion of daily dry matter intake, these results represent the marginal contribution and potential risk posed solely by the *hora* resource, rather than a complete net dietary mineral balance. Additionally, the sample collection was conducted exclusively during the dry season (December-February), which typically represents the period of peak mineral concentration. During the wet season, increased precipitation likely dilutes the concentration of total dissolved solids and specific minerals, which may improve palatability while reducing the net mineral density consumed. Moreover, availability of surface puddles or rainwater during the rainy season could lead to a voluntary reduction in *hora* consumption, resulting in significant seasonal variations in daily mineral intake that were not captured in this cross-sectional study.

Finally, it is crucial to distinguish between gross intake and bioavailable intake. The actual biological utility of these minerals is likely constrained by the alkaline soil pH (up to 8.28), which is not conducive to optimal gastrointestinal absorption [46]. Furthermore, the excessive levels of Fe, Mo, and Mn identified in the present study induce secondary Cu deficiency through strong antagonistic interactions [28]. In the rumen environment, high concentrations of Mo and S favor the formation of thiomolybdates; these compounds bind with copper to form insoluble complexes, rendering it unavailable for systemic absorption even when gross copper intake appears sufficient [47,48]. Consequently, strategic supplementation with *hora* must be integrated into a holistic nutritional management plan. Such plans must considers the specific zootechnical purpose of the animals and optimizes delivery methods to maximize mineral balance while preventing the risks of toxicity or palatability-induced dehydration.

## Conclusion

The physicochemical properties and mineral concentration of *hora* and mineral-enriched surrounding soils in the study area show significant district-level variation. While most physicochemical properties and mineral concentrations in *hora* were generally within an acceptable range for cattle, there are concerns about elevated levels of EC, TDS, and NH_3_. *Hora* water serves as a highly valuable and cost-effective source of Na and Se, particularly in regions where dietary Na deficiency is prevalent. The mineral-enriched surrounding soils, while offering potential as a natural mineral supplement source, present risks due to high concentration of antagonistic elements like Fe, Mo, and Mn that exceeded MTL. To fully optimize these indigenous resources, it is essential to move toward managed access, improved infrastructure, and the exploration of value-added products like mineral premixes, alongside community-based educational initiatives for livestock owners. Future research must build upon these findings by prioritizing landscape-level comparative studies that include soil, water, and background forage samples collected from sites distal to the *hora* points. Furthermore, investigating the bioavailability of these minerals and determining actual ingestion rates will be crucial for moving from theoretical risk modeling to precise nutritional management strategies for cattle.

## Supporting information

S1 File Raw data of mineral concentrations of *hora* mineral water. (XLSX)

S2 File Raw data of mineral concentrations of soil. (XLSX)

S3 File Raw data used to estimate mineral contribution. (XLSX)

S4 File R script used for data analysis. (R)

## References

[1] Quattrini S, Pampaloni B, Brandi ML. Natural mineral waters: chemical characteristics and health effects. Clin Cases Miner Bone Metab. 2016;13(3):173–80. doi: 10.11138/ccmbm/2016.13.3.173 28228777 PMC5318167

[2] Pampaloni B, Brandi ML. Mineral water as food for bone: an overview. Int J Bone Fragil. 2022;2(2):48–55. doi: 10.57582/ijbf.220202.048

[3] Kroesen LP, Hik DS, Cherry SG. Patterns of decadal, seasonal and daily visitation to mineral licks, a critical resource hotspot for mountain goats Oreamnos americanus in the Rocky Mountains. Wildl Biol. 2020;2020(4):1–11. doi: 10.2981/wlb.00736

[4] Mosquera D, Vinueza Hidalgo G, Blake J. Patterns of mineral lick visitation by Linnaeus’s two-toed sloth Choloepus didactylus (Pilosa, Megalonychidae) in eastern Ecuador. Notas Mam Sud. 2019;01(1):001–11. doi: 10.31687/saremnms.19.0.07

[5] Razali NB, Shafie MSH, Mohd Jobran RA, Abdul Karim NH, Khamis S, Mohd-Taib FS, et al. Physical factors at salt licks influenced the frequency of wildlife visitation in the Malaysian tropical rainforest. Trop Zool. 2020;33(3). doi: 10.4081/tz.2020.69

[6] Alemu T, Mulugeta E, Tadese M. Determination of physicochemical parameters of “Hora” natural mineral water and soil in Senkele Kebele, Oromia Region, Ethiopia. Cogent Chem. 2017;3(1):1354800. doi: 10.1080/23312009.2017.1354800

[7] Edae M, Dirribsa W, Mitiku T. Hora and Cattle owners of Maccaa Oromo in Ethiopia: an analysis from folkloric perspective. IJMMU. 2017;4(4):40. doi: 10.18415/ijmmu.v4i4.87

[8] Kenea AM, Tolemariam Ejeta T, Duguma Iticha B, Dierenfeld ES, Paul Jules Janssens G, Demeke Cherkos S. Natural mineral spring water (hora) and surrounding soils in southwestern Ethiopia: farmers’ feeding practices and their perception about its nutritional roles on animal performance. Heliyon. 2024;10(12):e33299. doi: 10.1016/j.heliyon.2024.e33299 39027454 PMC11254596

[9] Sim SF, Azlan JM, Abdul N, Rahman SL, Kang PL. Mineral characteristics of tropical salt licks in Sarawak, the Northwest of Borneo Island. J Sustain Sci Manag. 2020;15:53–62. doi: 10.46754/jssm.2020.12.005

[10] Lazarus BA, Che-Amat A, Abdul Halim Shah MM, Hamdan A, Abu Hassim H, Mustaffa Kamal F, et al. Impact of natural salt lick on the home range of Panthera tigris at the Royal Belum Rainforest, Malaysia. Sci Rep. 2021;11(1):10596. doi: 10.1038/s41598-021-89980-0 34012045 PMC8134436

[11] Maro A, Dudley R. Non‐random distribution of ungulate salt licks relative to distance from North American oceanic margins. J Biogeogr. 2022;49(2):254–60. doi: 10.1111/jbi.14312

[12] Lee JM, Koh D-C, Chae G-T, Kee W-S, Ko K-S. Integrated assessment of major element geochemistry and geological setting of traditional natural mineral water sources in South Korea at the national scale. J Hydrol. 2021;598:126249. doi: 10.1016/j.jhydrol.2021.126249

[13] Li Vigni L, Daskalopoulou K, Calabrese S, Kyriakopoulos K, Bellomo S, Brusca L, et al. Characterization of trace elements in thermal and mineral waters of Greece. Environ Sci Pollut Res Int. 2023;30(32):78376–93. doi: 10.1007/s11356-023-27829-x 37268809 PMC10313562

[14] Chiodi M. The distribution, properties and uses of mineral springs in the Harenna forest. Walia. 2011;2011:225–42.

[15] Desalegn T, Mohammed YK. Physical properties and critical mineral concentration of mineral waters commonly consumed by camels (*Camelus dromedarius*) in Jijiga District, Eastern Ethiopia. LRRD. 2012;24:1–8.

[16] Zeleke M, Kechero Y, Kurtu MY. Practice of local mineral supplementation to livestock’s and perception of farmer’s in Humbo woreda, Wolaita zone, Ethiopia. Glob Vet. 2016;17:114–21. doi: 10.5829/idosi.gv.2016.17.02.10415

[17] Desalegn T, Tolera A, Nurfeta A, Engle T. Indigenous mineral supplements of livestock and farmers’ perception on the supplements in Wolaita lowlands, southern Ethiopia. Anim Prod. 2018;18:74–87.

[18] Georgievskii VI, Annenkov BN, Samokhin VT. Mineral nutrition of animals: studies in the agricultural and food sciences. Elsevier; 2013.

[19] Suttle N. Mineral status of livestock. In: Mineral nutrition of livestock. 5th ed. CABI; 2022. p. 38–54.

[20] Saha SK, Pathak NN. Mineral nutrition. In: Fundamentals of animal nutrition. Singapore: Springer; 2021. p. 113–31. doi: 10.1007/978-981-15-9125-9_9

[21] Razzaque MS, Wimalawansa SJ. Minerals and Human health: from deficiency to toxicity. Nutrients. 2025;17(3):454. doi: 10.3390/nu17030454 39940312 PMC11820417

[22] A P H A. Standard methods for the examination of water and wastewater. 23 ed. Washington, DC: American Public Health Association; 2017.

[23] Zanetti D, Prados LF, Menezes ACB, Silva BC, Pacheco MVC, Silva FAS, et al. Prediction of water intake to Bos indicus beef cattle raised under tropical conditions. J Anim Sci. 2019;97(3):1364–74. doi: 10.1093/jas/skz003 30753494 PMC6396249

[24] McConnachie S, Clayton E, Arundell L, Dominiak BC, Brock P. How much soil do cattle ingest? A review. Anim Prod Sci. 2024;64(15). doi: 10.1071/an24130

[25] NRC. Nutrient requirements of beef cattle. In: Eighth Revised Edition ed. Washington, D.C.: National Academies Press; 2016. doi: 10.17226/19014 38386771

[26] R Core Team. R: a language and environment for statistical computing. Available from: https://www.r-project.org/. 2024.

[27] CCME. Canadian water quality guidelines for the protection of agricultural water uses. Canadian Council of Ministers of the Environment; 2005. Available from: https://ccme.ca/en/resources# 10.1006/rtph.1994.1074 7724832

[28] López-Alonso M. Trace minerals and livestock: not too much not too little. ISRN Vet Sci. 2012;2012:704825. doi: 10.5402/2012/704825 23762589 PMC3671743

[29] Patra AK, Dos Santos Ribeiro LP, Yirga H, Sonibare AO, Askar AR, Hussein AH, et al. Effects of the concentration and nature of total dissolved solids in drinking water on feed intake, nutrient digestion, energy balance, methane emission, ruminal fermentation, and blood constituents in different breeds of young goats and hair sheep. Anim Nutr. 2023;16:84–95. doi: 10.1016/j.aninu.2023.10.002 38333574 PMC10851211

[30] Moehlenpah AN. Water and forage intake, diet digestibility, and blood parameters for beef cows and growing heifers consuming water with varying concentrations of total dissolved salts. Oklahoma State University; 2020. Available from: https://search.proquest.com/openview/b9dfad22772afa33066b88dd7c267671/1?pq-origsite=gscholar&cbl=18750&diss=y 10.1093/jas/skab282 PMC8557798 34618893

[31] Moehlenpah AN, Ribeiro LPS, Puchala R, Goetsch AL, Beck P, Pezeshki A, et al. Water and forage intake, diet digestibility, and blood parameters of beef cows and heifers consuming water with varying concentrations of total dissolved salts. J Anim Sci. 2021;99(10):skab282. doi: 10.1093/jas/skab282 34618893 PMC8557798

[32] Khanna S, Potliya S, Ganguly A, Singh H, Maharana BR, Singh D, et al. An Assessment of Indian livestock owners’ selective management practices for risk amelioration of obstructive urolithiasis in water buffalo male calves. AJDFR. 2024. doi: 10.18805/ajdfr.dr-2244

[33] Shammi M, Rahman MM, Bondad SE, Bodrud-Doza M. Impacts of salinity intrusion in community health: a review of experiences on drinking water sodium from coastal areas of Bangladesh. Healthcare (Basel). 2019;7(1):50. doi: 10.3390/healthcare7010050 30909429 PMC6473225

[34] Boyd CE. Suspended solids, color, turbidity, and light. In: Water quality: an introduction. Cham: Springer International Publishing; 2020. p. 119–33. doi: 10.1007/978-3-030-23335-8_6

[35] Mann AG, Tam CC, Higgins CD, Rodrigues LC. The association between drinking water turbidity and gastrointestinal illness: a systematic review. BMC Public Health. 2007;7:256. doi: 10.1186/1471-2458-7-256 17888154 PMC2174477

[36] Senevirathne ND, Anderson JL, Erickson PS, Rovai M. Evaluation of the effects of water quality on drinking preferences of heifer calves. JDS Commun. 2021;2(6):393–7. doi: 10.3168/jdsc.2021-0101 36337115 PMC9623628

[37] Igwaran A, Kayode AJ, Moloantoa KM, Khetsha ZP, Unuofin JO. Cyanobacteria harmful algae blooms: Causes, impacts, and risk management. Water Air Soil Pollut. 2024;235:71. doi: 10.1007/s11270-023-06782-y

[38] WHO. Guidelines for drinking-water quality. 4th ed. Geneva: World Health Organization; 2017. Available from: https://iris.who.int/handle/10665/254637 28759192

[39] Woolway RI, Sharma S, Smol JP. Lakes in hot water: the impacts of a changing climate on aquatic ecosystems. Bioscience. 2022;72(11):1050–61. doi: 10.1093/biosci/biac052 36325103 PMC9618276

[40] Albertini MC, Dacha M, Teodori L, Conti ME. Drinking mineral waters: biochemical effects and health implications the state-of-the-art. IJENVH. 2007;1(1):153. doi: 10.1504/ijenvh.2007.012230

[41] Yirga H, Urge M, Goetsch AL, Tolera A, Puchala R, Patra AK. Effects of Salinity levels of drinking water on water intake and loss, feed utilization, body weight, thermoregulatory traits, and blood constituents in growing and mature blackhead ogaden sheep and somali goats. Animals (Basel). 2024;14(11):1565. doi: 10.3390/ani14111565 38891612 PMC11171153

[42] Neckermann K, Kechero Y. Mineral status of Zebu cattle (Bos indicus) in the Ethiopian rift valley. Ghent: Ghent University; 2017. Available from: https://libstore.ugent.be/fulltxt/RUG01/002/376/770/RUG01-002376770_2017_0001_AC.pdf

[43] Han WX, Fang JY, Reich PB, Ian Woodward F, Wang ZH. Biogeography and variability of eleven mineral elements in plant leaves across gradients of climate, soil and plant functional type in China. Ecol Lett. 2011;14(8):788–96. doi: 10.1111/j.1461-0248.2011.01641.x 21692962

[44] Suttle N. The mineral nutrition of livestock. 4th ed. Wallingford: CABI publ; 2010.

[45] Dermauw V, Yisehak K, Belay D, Van Hecke T, Du Laing G, Duchateau L, et al. Mineral deficiency status of ranging zebu (Bos indicus) cattle around the Gilgel Gibe catchment, Ethiopia. Trop Anim Health Prod. 2013;45(5):1139–47. doi: 10.1007/s11250-012-0337-4 23254979

[46] McDowell LR, Arthington JD. Minerals for grazing ruminants in tropical regions. 2005 [cited 17 Feb 2025]. Available from: https://www.cabidigitallibrary.org/doi/full/10.5555/20073126802

[47] Gould L, Kendall NR. Role of the rumen in copper and thiomolybdate absorption. Nutr Res Rev. 2011;24(2):176–82. doi: 10.1017/S0954422411000059 22296933 PMC3269883

[48] Conti RMC, da Silva TH, da Silva Brandão Guimarães IC, Bezerra HVA, Saran Netto A, Rodrigues PHM, et al. Influence of molybdenum and organic sources of copper and sulfur on the performance, carcass traits, blood mineral concentration, and ceruloplasmin activity in lambs. Animals (Basel). 2023;13(18):2945. doi: 10.3390/ani13182945 37760345 PMC10525667

