# Supplementary material for: Nutritional value of hora and mineral-enriched soil for cattle: Evaluation of mineral composition and physicochemical properties in southwestern Ethiopia
Source: PLoS One. 2026 May 14;21(5):e0348764. doi: 10.1371/journal.pone.0348764 (PMC13175342; doi:10.1371/journal.pone.0348764)
Supplement: Attachment — Submitted filename: PLOS ONE-Reviewer Report.doc [file pone.0348764.s005.doc]

**Reviewer Comments to the Authors**

**Manuscript Number:** PONE-D-25-68057

**Title:** Nutritional Value of Hora and Mineral-enriched Soil for Cattle: Evaluation of Mineral Composition and Physicochemical Properties in Southwestern Ethiopia.

**Decision:** Minor Revision

**General Evaluation** This study provides a valuable quantitative assessment of a traditional livestock feeding practice by evaluating the mineral composition and physicochemical properties of "hora" (natural mineral water) and its surrounding mineral-enriched soils in southwestern Ethiopia. By analyzing samples from the Bedele, Dabo, Gechi, and Borecha districts, the authors effectively highlight the potential of these resources as a strategic natural source of sodium and selenium, while also correctly identifying the toxicological risks associated with excessive iron, molybdenum, and manganese in the soil.

The methodology, incorporating APHA standards for water and ICP-OES analysis for mineral quantification, is technically sound. Furthermore, modeling the potential daily mineral intake based on established water and dry matter consumption formulas for tropical cattle is a highly commendable approach that bridges analytical chemistry with practical animal nutrition.

The manuscript aligns well with the publication criteria of PLOS ONE, presenting original research with rigorously supported conclusions. However, addressing a few methodological limitations and contextual factors in the discussion will significantly strengthen the final manuscript.

**Specific Comments**

1. **Baseline and Control Data:** As the authors rightly acknowledged in the discussion, the study currently lacks a comparative baseline from sites distal to the hora springs. Without these control samples for background water and soil, quantifying the exact magnitude of mineral enrichment relative to the region's basal environmental status remains difficult. I recommend expanding the discussion slightly to address how this limitation might influence the interpretation of the net dietary mineral balance.
2. **Seasonal Variability:** The sampling was exclusively conducted during the dry season (December-February). It is highly probable that precipitation during the wet season would dilute the total dissolved solids (TDS) and alter specific mineral concentrations. Please add a brief paragraph discussing the potential impact of seasonal dynamics on water palatability, mineral concentration, and consequently, the variations in the cattle's daily mineral intake throughout the year.
3. **Bioavailability vs. Gross Intake:** The daily soil ingestion was reasonably estimated at 0.5 kg DM/hd/day. While the gross mineral intake calculations presented in Table 4 are helpful , the alkaline pH recorded in the soil samples (e.g., reaching up to 8.1 in Bedele and 8.28 in Dabo) does not favor the optimal absorption of most minerals in the gastrointestinal tract. Furthermore, the extremely high levels of iron, molybdenum, and manganese could induce secondary copper deficiency due to strong antagonistic interactions. I suggest explicitly emphasizing that the *calculated* gross intake does not equal the *bioavailable* intake, particularly under these alkaline and highly antagonistic matrix conditions.
4. **Methodological Clarity (HG-ICP-OES):** The use of microwave digestion and subsequent hydride generation (HG-ICP-OES) for selenium determination is an excellent methodological choice to enhance sensitivity and minimize matrix interferences. To further validate this analytical rigor, please consider adding a brief statement in the methodology section regarding the recovery rates or the limits of quantification (LOQ) specific to this selenium analysis

**Comments to the Editor**

Dear Editor,

This manuscript presents a solid, quantitative evaluation of a traditional livestock feeding practice in Ethiopia. The methodology used for mineral analysis and the subsequent modeling of daily mineral intake for cattle are robust and highly relevant to the field of animal nutrition. The authors have done a commendable job bridging analytical chemistry with practical livestock management, and the study clearly aligns with PLOS ONE's criteria for publishing technically sound, original research.

My recommendation is "Minor Revision." The core science and data are reliable. The revisions I have requested are primarily aimed at improving the depth of the discussion—specifically regarding the critical distinction between gross mineral intake and actual bioavailability (given the alkaline soil pH and potential antagonistic mineral interactions), as well as acknowledging the limitations regarding baseline/control data and seasonal variability. I have also asked for a minor methodological clarification regarding the LOQ/recovery rates for the selenium analysis to ensure full transparency.

I believe the authors can readily address these points. Once revised, this manuscript will be a valuable and rigorous addition to the literature.
