# Supplementary material for: Nutritional value of hora and mineral-enriched soil for cattle: Evaluation of mineral composition and physicochemical properties in southwestern Ethiopia
Source: PLoS One. 2026 May 14;21(5):e0348764. doi: 10.1371/journal.pone.0348764 (PMC13175342; doi:10.1371/journal.pone.0348764)
Supplement: Attachment — Submitted filename: Response to Reviewers.docx [file pone.0348764.s007.docx]

**Authors’ Response to Editor and Reviewers’ Comments**

**Manuscript Title**: Nutritional Value of Hora and Mineral-Enriched Soil for Cattle: Evaluation of Mineral Composition and Physicochemical Properties in Southwestern Ethiopia

**Manuscript ID**: PONE-D-25-68057

**Dear Academic Editor and Reviewers,**

Thank you for the opportunity to revise and resubmit our manuscript. We appreciate the constructive and valuable feedback, which has significantly improved the quality of our work. We have carefully addressed all comments raised by the Academic Editor and the Reviewers, incorporating the suggested changes into the revised manuscript. A marked-up copy highlighting all revisions is provided as the 'Revised Manuscript with Track Changes', and a clean version is provided as the 'Manuscript'.

**Sincerely,**

Dr. Ashenafi Miresa Kenea (Corresponding Author)

April 15, 2026

**Response to Academic Editor**

**Comment 1**: Expand the discussion on the absence of control samples (areas distant from the “hora” sources), highlighting how this limitation may influence the interpretation of the degree of mineral enrichment.

**Author Response:** We have added a new subsection (**Limitations and Future Directions)** in the Discussion (**Lines 326-342, page 16-17**) acknowledging the lack of distal control samples. We explicitly discuss how this affects the interpretation of enrichment magnitude relative to the basal environmental status of the region.

**Comment 2**: Include a paragraph addressing the potential impact of seasonality (especially the rainy season) on mineral concentrations, total dissolved solids, and animal intake.

**Author Response**: A paragraph has been added to the discussion **(Lines 335-342, page 17)** addressing seasonal dynamics. We discuss how rainy-season precipitation likely dilutes TDS, potentially altering mineral concentrations and cattle intake behavior compared to the dry-season sampling period.

**Comment 3**: Explicitly emphasize that the estimated mineral intake values represent gross intake rather than bioavailable intake, considering: (1) the alkaline soil pH, which may reduce absorption; and (2) possible antagonistic interactions among minerals (e.g., Fe, Mo, and Mn affecting copper metabolism).

**Author Response:** We have updated the discussion (**Lines 343-345, page 17)** to explicitly state that the calculated values represent gross intake rather than bioavailable intake. We have also added a mechanistic discussion regarding the limiting effects of alkaline pH and mineral antagonisms specifically the formation of thiomolybdates involving Fe, Mo, and Mn on copper metabolism.

**Comment 4**: Provide additional information on the selenium analysis by HG-ICP-OES, particularly limits of quantification (LOQ) and/or recovery rates, to strengthen analytical transparency and robustness.

**Author Response:** We have updated the Materials and Methods section **(Lines 161-171, page 6)** to include comprehensive instrumental parameters, including plasma power (1400 W), gas flow rates, and calibration protocols for the Arcos_SOP-ICP-OES (Model FSH-12-2010). While specific batch-recovery rates were not indexed in the lab report, we have clarified that the method was within the validated linear range through daily standardization using high-purity stock solutions (Inorganic Ventures, Germany) to ensure sensitivity and accuracy within manufacturer-optimized specifications.

**Comment 5**: Conduct a general grammatical revision of the manuscript. Standardize citations and references, correcting the numbering throughout the text (e.g., adjust “CCME (2025)” to the appropriate format, such as “CCME [27]”). Review punctuation and formatting in the indicated lines (p. 5, lines 129–130). Correctly format chemical notation (e.g., use subscript in NH₃).

**Author Response:** A full grammatical review was conducted. Citations have been standardized to the [Bracketed Number] format required by PLOS ONE. Punctuation on page 5 has been corrected, and all chemical notations have been properly formatted with subscripts and superscripts throughout the manuscript.

**Response to Journal Requirements**:

- As per Requirement 2, we have added a statement to the Methods section (**Lines 204-208, page 7)** clarifying that no specific field permits were required as the sites are public traditional grazing areas.
- In accordance with data availability requirement, we have uploaded our raw measurement data as 'S1_Data.xlsx' to ensure the minimal data set is available for reproducibility.
- We have provided the author-generated R code used for statistical analysis and mineral intake modeling as 'S1_Code.R'.

**Reviewer Comments**

**Reviewer #1: General Evaluation**

This study provides a valuable quantitative assessment of a traditional livestock feeding practice by evaluating the mineral composition and physicochemical properties of "hora" (natural mineral water) and its surrounding mineral-enriched soils in southwestern Ethiopia. By analyzing samples from the Bedele, Dabo, Gechi, and Borecha districts, the authors effectively highlight the potential of these resources as a strategic natural source of sodium and selenium, while also correctly identifying the toxicological risks associated with excessive iron, molybdenum, and manganese in the soil.

The methodology, incorporating APHA standards for water and ICP-OES analysis for mineral quantification, is technically sound. Furthermore, modeling the potential daily mineral intake based on established water and dry matter consumption formulas for tropical cattle is a highly commendable approach that bridges analytical chemistry with practical animal nutrition. The manuscript aligns well with the publication criteria of PLOS ONE, presenting original research with rigorously supported conclusions. However, addressing a few methodological limitations and contextual factors in the discussion will significantly strengthen the final manuscript.

**Author Response:** We thank the reviewer for recognizing the value of this quantitative assessment and for the positive comments regarding our methodology and the bridge between analytical chemistry and animal nutrition.

**Specific Comments**

**Baseline and Control Data**: As the authors rightly acknowledged in the discussion, the study currently lacks a comparative baseline from sites distal to the hora springs. Without these control samples for background water and soil, quantifying the exact magnitude of mineral enrichment relative to the region's basal environmental status remains difficult. I recommend expanding the discussion slightly to address how this limitation might influence the interpretation of the net dietary mineral balance.

**Response:** We have expanded the Methodological limitation section of the discussion. We now explicitly acknowledge that without distal control samples, we cannot precisely quantify the exact magnitude of enrichment relative to the region's basal environmental status. This addition clarifies that our findings represent the specific characteristics of the *hora* sites rather than a regional comparison.

**Seasonal Variability**: The sampling was exclusively conducted during the dry season (December-February). It is highly probable that precipitation during the wet season would dilute the total dissolved solids (TDS) and alter specific mineral concentrations. Please add a brief paragraph discussing the potential impact of seasonal dynamics on water palatability, mineral concentration, and consequently, the variations in the cattle's daily mineral intake throughout the year.

**Author Response:** We have added a new paragraph to the subsection **Limitations and Future Directions** at the end of discussion and addressing seasonal variations **(Line 337-342, page 17).** We acknowledge that dry-season sampling may present peak concentrations and discuss how rainy-season precipitation may dilute minerals and influence the cattle’s water source preference and daily mineral intake patterns.

**Bioavailability vs. Gross Intake**: The daily soil ingestion was reasonably estimated at 0.5 kg DM/hd/day. While the gross mineral intake calculations presented in Table 4 are helpful, the alkaline pH recorded in the soil samples (e.g., reaching up to 8.1 in Bedele and 8.28 in Dabo) does not favor the optimal absorption of most minerals in the gastrointestinal tract. Furthermore, the extremely high levels of iron, molybdenum, and manganese could induce secondary copper deficiency due to strong antagonistic interactions. I suggest explicitly emphasizing that the calculated gross intake does not equal the bioavailable intake, particularly under these alkaline and highly antagonistic matrix conditions.

**Author Response:** We have updated the discussion and the description of Table 4 to emphasize that values represent gross intake. We added text explaining that the recorded alkaline pH (reaching 8.28 in Dabo) likely hinders absorption and discussed the risk of secondary copper deficiency caused by antagonistic interactions involving Fe, Mo, and Mn. Furthermore, we now discuss the risk of secondary copper deficiency caused by the antagonistic interactions of the extremely high levels of Fe, Mo, and Mn identified in our soil analysis.

**Methodological Clarity (HG-ICP-OES):** The use of microwave digestion and subsequent hydride generation (HG-ICP-OES) for selenium determination is an excellent methodological choice to enhance sensitivity and minimize matrix interferences. To further validate this analytical rigor, please consider adding a brief statement in the methodology section regarding the recovery rates or the limits of quantification (LOQ) specific to this selenium analysis.

**Author Response:** We agree that analytical transparency is vital. While specific recovery rates were not indexed in the final lab report, we have updated the Materials and Methods to reflect the instrumental optimization (daily standardization and calibration using Inorganic Ventures stock solutions) and adherence to standard manufacturer protocols for trace element sensitivity.

**Reviewer #2: Editor**

I appreciate the trust placed in me to review the article titled: “Nutritional Value of Alfalfa and Mineral-Enriched Soil for Livestock: Evaluation of Mineral Composition and Physicochemical Properties in Southwest Ethiopia.” The article is well-structured, and the study’s objective is relevant.

**Author Response:** We thank the Reviewer for their appreciative comments regarding the structure and relevance of our study. We note that the Reviewer's summary mentioned 'Alfalfa'; however, we would like to clarify that our study focus and manuscript title remain: **Nutritional Value of Hora and Mineral-enriched Soil for Cattle: Evaluation of Mineral Composition and Physicochemical Properties in Southwestern Ethiopia.**

Some minor grammatical details have been identified. Regarding the citation of references within the document, a careful review is necessary, and the numbering should be restructured. The inconsistencies begin at the foot of Table 1, where it appears as: CCME (2025) and should be CCME [27].

**Author Response:** We have restructured the numbering of references to strictly follow PLOS ONE style. Specifically, in Table 1, the citation "CCME (2025)" has been corrected to the bracketed format: **CCME [27]**.

Other grammatical details are observed on page 5, lines 129, 130 Please review the punctuation and subscript in the chemical formula NH3, line 132.

**Author Response**: We have conducted a thorough review of the punctuation and formatting on page 5. Chemical notations have been corrected to include proper subscripts consistently throughout the manuscript.
