# Supplementary material for: Nutritional value of hora and mineral-enriched soil for cattle: Evaluation of mineral composition and physicochemical properties in southwestern Ethiopia
Source: PLoS One. 2026 May 14;21(5):e0348764. doi: 10.1371/journal.pone.0348764 (PMC13175342; doi:10.1371/journal.pone.0348764)
Supplement: Peer review history

# Peer review: Decision Letter 0

**DOI:** 10.1371/journal.pone.0348764.r001

**Authors:** Elma Izze Magalhães

6 Apr 2026

PONE-D-25-68057

Nutritional Value of Hora and Mineral-enriched Soil for Cattle: Evaluation of Mineral Composition and Physicochemical Properties in Southwestern Ethiopia.

PLOS One

Dear Dr. Miresa,

Thank you for submitting your manuscript to PLOS ONE. After careful consideration, we feel that it has merit but does not fully meet PLOS ONE’s publication criteria as it currently stands. Therefore, we invite you to submit a revised version of the manuscript that addresses the points raised during the review process.

==============================

ACADEMIC EDITOR:

Changes  required for acceptance:

- Expand the discussion on the absence of control samples (areas distant from the “hora” sources), highlighting how this limitation may influence the interpretation of the degree of mineral enrichment.

- Include a paragraph addressing the potential impact of seasonality (especially the rainy season) on mineral concentrations, total dissolved solids, and animal intake.

- Explicitly emphasize that the estimated mineral intake values represent gross intake rather than bioavailable intake, considering: (1) the alkaline soil pH, which may reduce absorption; and (2) possible antagonistic interactions among minerals (e.g., Fe, Mo, and Mn affecting copper metabolism).

- Provide additional information on the selenium analysis by HG-ICP-OES, particularly limits of quantification (LOQ) and/or recovery rates, to strengthen analytical transparency and robustness.

- Conduct a general grammatical revision of the manuscript.

- Standardize citations and references, correcting the numbering throughout the text (e.g., adjust “CCME (2025)” to the appropriate format, such as “CCME [27]”).

- Review punctuation and formatting in the indicated lines (p. 5, lines 129–130).

- Correctly format chemical notation (e.g., use subscript in NH₃).

==============================

Please submit your revised manuscript by May 22 2026 11:59PM. If you will need more time than this to complete your revisions, please reply to this message or contact the journal office at plosone@plos.org. When you're ready to submit your revision, log on to https://www.editorialmanager.com/pone/ and select the ’submissions Needing Revision' folder to locate your manuscript file.

Please include the following items when submitting your revised manuscript:

- A letter that responds to each point raised by the academic editor and reviewer(s). You should upload this letter as a separate file labeled 'Response to Reviewers'.
- A marked-up copy of your manuscript that highlights changes made to the original version. You should upload this as a separate file labeled 'Revised Manuscript with Track Changes'.
- An unmarked version of your revised paper without tracked changes. You should upload this as a separate file labeled 'Manuscript'.

If you would like to make changes to your financial disclosure, please include your updated statement in your cover letter. Guidelines for resubmitting your figure files are available below the reviewer comments at the end of this letter.

If applicable, we recommend that you deposit your laboratory protocols in protocols.io to enhance the reproducibility of your results. Protocols.io assigns your protocol its own identifier (DOI) so that it can be cited independently in the future. For instructions see: https://journals.plos.org/plosone/s/submission-guidelines#loc-laboratory-protocols. Additionally, PLOS ONE offers an option for publishing peer-reviewed Lab Protocol articles, which describe protocols hosted on protocols.io. Read more information on sharing protocols at https://plos.org/protocols?utm_medium=editorial-email&utm_source=authorletters&utm_campaign=protocols.

As the corresponding author, your ORCID iD is verified in the submission system and will appear in the published article. PLOS supports the use of ORCID, and we encourage all coauthors to register for an ORCID iD and use it as well. Please encourage your coauthors to verify their ORCID iD within the submission system before final acceptance, as unverified ORCID iDs will not appear in the published article. Only the individual author can complete the verification step; PLOS staff cannot verify ORCID iDs on behalf of authors.

We look forward to receiving your revised manuscript.

Kind regards,

Elma Izze Da Silva Magalhães

Academic Editor

PLOS One

Journal Requirements:

When submitting your revision, we need you to address these additional requirements.

1. Please ensure that your manuscript meets PLOS ONE’s style requirements, including those for file naming. The PLOS ONE style templates can be found at

https://journals.plos.org/plosone/s/file?id=wjVg/PLOSOne_formatting_sample_main_body.pdf and

https://journals.plos.org/plosone/s/file?id=ba62/PLOSOne_formatting_sample_title_authors_affiliations.pdf

2. In your Methods section, please provide additional information regarding the permits you obtained for the work. Please ensure you have included the full name of the authority that approved the field site access and, if no permits were required, a brief statement explaining why

3. Please note that PLOS One has specific guidelines on code sharing for submissions in which author-generated code underpins the findings in the manuscript. In these cases, we expect all author-generated code to be made available without restrictions upon publication of the work. Please review our guidelines at https://journals.plos.org/plosone/s/materials-and-software-sharing#loc-sharing-code and ensure that your code is shared in a way that follows best practice and facilitates reproducibility and reuse.

4. We note that your Data Availability Statement is currently as follows: "All relevant data are within the manuscript and its Supporting Information files."

Please confirm at this time whether or not your submission contains all raw data required to replicate the results of your study. Authors must share the “minimal data set” for their submission. PLOS defines the minimal data set to consist of the data required to replicate all study findings reported in the article, as well as related metadata and methods (https://journals.plos.org/plosone/s/data-availability#loc-minimal-data-set-definition).

For example, authors should submit the following data:

- The values behind the means, standard deviations and other measures reported;

- The values used to build graphs;

- The points extracted from images for analysis.

Authors do not need to submit their entire data set if only a portion of the data was used in the reported study.

If your submission does not contain these data, please either upload them as Supporting Information files or deposit them to a stable, public repository and provide us with the relevant URLs, DOIs, or accession numbers. For a list of recommended repositories, please see https://journals.plos.org/plosone/s/recommended-repositories.

If there are ethical or legal restrictions on sharing a de-identified data set, please explain them in detail (e.g., data contain potentially sensitive information, data are owned by a third-party organization, etc.) and who has imposed them (e.g., an ethics committee). Please also provide contact information for a data access committee, ethics committee, or other institutional body to which data requests may be sent. If data are owned by a third party, please indicate how others may request data access.

5. If the reviewer comments include a recommendation to cite specific previously published works, please review and evaluate these publications to determine whether they are relevant and should be cited. There is no requirement to cite these works unless the editor has indicated otherwise.

6. Please review your reference list to ensure that it is complete and correct. If you have cited papers that have been retracted, please include the rationale for doing so in the manuscript text, or remove these references and replace them with relevant current references. Any changes to the reference list should be mentioned in the rebuttal letter that accompanies your revised manuscript. If you need to cite a retracted article, indicate the article’s retracted status in the References list and also include a citation and full reference for the retraction notice.

Reviewers' comments:

Reviewer’s Responses to Questions

**Comments to the Author**

1. Is the manuscript technically sound, and do the data support the conclusions?

The manuscript must describe a technically sound piece of scientific research with data that supports the conclusions. Experiments must have been conducted rigorously, with appropriate controls, replication, and sample sizes. The conclusions must be drawn appropriately based on the data presented.

Reviewer #1: Yes

Reviewer #2: Yes

**********

2. Has the statistical analysis been performed appropriately and rigorously?

Reviewer #1: Yes

Reviewer #2: Yes

**********

3. Have the authors made all data underlying the findings in their manuscript fully available?

The PLOS Data policy requires authors to make all data underlying the findings described in their manuscript fully available without restriction, with rare exception (please refer to the Data Availability Statement in the manuscript PDF file). The data should be provided as part of the manuscript or its supporting information, or deposited to a public repository. For example, in addition to summary statistics, the data points behind means, medians and variance measures should be available. If there are restrictions on publicly sharing data—e.g. participant privacy or use of data from a third party—those must be specified.

Reviewer #1: Yes

Reviewer #2: Yes

**********

4. Is the manuscript presented in an intelligible fashion and written in standard English?

PLOS ONE does not copyedit accepted manuscripts, so the language in submitted articles must be clear, correct, and unambiguous. Any typographical or grammatical errors should be corrected at revision, so please note any specific errors here.

Reviewer #1: Yes

Reviewer #2: Yes

**********

5. Review Comments to the Author

Please use the space provided to explain your answers to the questions above. You may also include additional comments for the author, including concerns about dual publication, research ethics, or publication ethics. (Please upload your review as an attachment if it exceeds 20,000 characters)

Reviewer #1: General Evaluation

This study provides a valuable quantitative assessment of a traditional livestock feeding practice by evaluating the mineral composition and physicochemical properties of "hora" (natural mineral water) and its surrounding mineral-enriched soils in southwestern Ethiopia. By analyzing samples from the Bedele, Dabo, Gechi, and Borecha districts, the authors effectively highlight the potential of these resources as a strategic natural source of sodium and selenium, while also correctly identifying the toxicological risks associated with excessive iron, molybdenum, and manganese in the soil.

The methodology, incorporating APHA standards for water and ICP-OES analysis for mineral quantification, is technically sound. Furthermore, modeling the potential daily mineral intake based on established water and dry matter consumption formulas for tropical cattle is a highly commendable approach that bridges analytical chemistry with practical animal nutrition. The manuscript aligns well with the publication criteria of PLOS ONE, presenting original research with rigorously supported conclusions. However, addressing a few methodological limitations and contextual factors in the discussion will significantly strengthen the final manuscript.

Specific Comments

Baseline and Control Data: As the authors rightly acknowledged in the discussion, the study currently lacks a comparative baseline from sites distal to the hora springs. Without these control samples for background water and soil, quantifying the exact magnitude of mineral enrichment relative to the region’s basal environmental status remains difficult. I recommend expanding the discussion slightly to address how this limitation might influence the interpretation of the net dietary mineral balance.

Seasonal Variability: The sampling was exclusively conducted during the dry season (December-February). It is highly probable that precipitation during the wet season would dilute the total dissolved solids (TDS) and alter specific mineral concentrations. Please add a brief paragraph discussing the potential impact of seasonal dynamics on water palatability, mineral concentration, and consequently, the variations in the cattle’s daily mineral intake throughout the year.

Bioavailability vs. Gross Intake: The daily soil ingestion was reasonably estimated at 0.5 kg DM/hd/day. While the gross mineral intake calculations presented in Table 4 are helpful, the alkaline pH recorded in the soil samples (e.g., reaching up to 8.1 in Bedele and 8.28 in Dabo) does not favor the optimal absorption of most minerals in the gastrointestinal tract. Furthermore, the extremely high levels of iron, molybdenum, and manganese could induce secondary copper deficiency due to strong antagonistic interactions. I suggest explicitly emphasizing that the calculated gross intake does not equal the bioavailable intake, particularly under these alkaline and highly antagonistic matrix conditions.

Methodological Clarity (HG-ICP-OES): The use of microwave digestion and subsequent hydride generation (HG-ICP-OES) for selenium determination is an excellent methodological choice to enhance sensitivity and minimize matrix interferences. To further validate this analytical rigor, please consider adding a brief statement in the methodology section regarding the recovery rates or the limits of quantification (LOQ) specific to this selenium analysis.

Reviewer #2: Editor

I appreciate the trust placed in me to review the article titled: “Nutritional Value of Alfalfa and Mineral-Enriched Soil for Livestock: Evaluation of Mineral Composition and Physicochemical Properties in Southwest Ethiopia.” The article is well-structured, and the study’s objective is relevant.

Some minor grammatical details have been identified. Regarding the citation of references within the document, a careful review is necessary, and the numbering should be restructured. The inconsistencies begin at the foot of Table 1, where it appears as: CCME (2025) and should be CCME [27].

Other grammatical details are observed on page 5, lines 129, 130 Please review the punctuation and subscript in the chemical formula NH3, line 132.

**********

6. PLOS authors have the option to publish the peer review history of their article (what does this mean?). If published, this will include your full peer review and any attached files.

If you choose “no”, your identity will remain anonymous but your review may still be made public.

**Do you want your identity to be public for this peer review?** For information about this choice, including consent withdrawal, please see our Privacy Policy.

Reviewer #1: No

Reviewer #2: No

**********

[NOTE: If reviewer comments were submitted as an attachment file, they will be attached to this email and accessible via the submission site. Please log into your account, locate the manuscript record, and check for the action link "View Attachments". If this link does not appear, there are no attachment files.]

To ensure your figures meet our technical requirements, please review our figure guidelines: https://journals.plos.org/plosone/s/figures

You may also use PLOS’s free figure tool, NAAS, to help you prepare publication quality figures: https://journals.plos.org/plosone/s/figures#loc-tools-for-figure-preparation.

NAAS will assess whether your figures meet our technical requirements by comparing each figure against our figure specifications.

Attachment Submitted filename: PLOS ONE-Reviewer Report.doc

# Author comment: Author response to Decision Letter 1

**DOI:** 10.1371/journal.pone.0348764.r002

16 Apr 2026

Response to Academic Editor

Comment 1: Expand the discussion on the absence of control samples (areas distant from the “hora” sources), highlighting how this limitation may influence the interpretation of the degree of mineral enrichment.

Author Response: We have added a new subsection (Limitations and Future Directions) in the Discussion (Lines 326-342, page 16-17) acknowledging the lack of distal control samples. We explicitly discuss how this affects the interpretation of enrichment magnitude relative to the basal environmental status of the region.

Comment 2: Include a paragraph addressing the potential impact of seasonality (especially the rainy season) on mineral concentrations, total dissolved solids, and animal intake.

Author Response: A paragraph has been added to the discussion (Lines 335-342, page 17) addressing seasonal dynamics. We discuss how rainy-season precipitation likely dilutes TDS, potentially altering mineral concentrations and cattle intake behavior compared to the dry-season sampling period.

Comment 3: Explicitly emphasize that the estimated mineral intake values represent gross intake rather than bioavailable intake, considering: (1) the alkaline soil pH, which may reduce absorption; and (2) possible antagonistic interactions among minerals (e.g., Fe, Mo, and Mn affecting copper metabolism).

Author Response: We have updated the discussion (Lines 343-345, page 17) to explicitly state that the calculated values represent gross intake rather than bioavailable intake. We have also added a mechanistic discussion regarding the limiting effects of alkaline pH and mineral antagonisms specifically the formation of thiomolybdates involving Fe, Mo, and Mn on copper metabolism.

Comment 4: Provide additional information on the selenium analysis by HG-ICP-OES, particularly limits of quantification (LOQ) and/or recovery rates, to strengthen analytical transparency and robustness.

Author Response: We have updated the Materials and Methods section (Lines 161-171, page 6) to include comprehensive instrumental parameters, including plasma power (1400 W), gas flow rates, and calibration protocols for the Arcos_SOP-ICP-OES (Model FSH-12-2010). While specific batch-recovery rates were not indexed in the lab report, we have clarified that the method was within the validated linear range through daily standardization using high-purity stock solutions (Inorganic Ventures, Germany) to ensure sensitivity and accuracy within manufacturer-optimized specifications.

Comment 5: Conduct a general grammatical revision of the manuscript. Standardize citations and references, correcting the numbering throughout the text (e.g., adjust “CCME (2025)” to the appropriate format, such as “CCME [27]”). Review punctuation and formatting in the indicated lines (p. 5, lines 129–130). Correctly format chemical notation (e.g., use subscript in NH₃).

Author Response: A full grammatical review was conducted. Citations have been standardized to the [Bracketed Number] format required by PLOS ONE. Punctuation on page 5 has been corrected, and all chemical notations have been properly formatted with subscripts and superscripts throughout the manuscript.

Response to Journal Requirements:

• As per Requirement 2, we have added a statement to the Methods section (Lines 204-208, page 7) clarifying that no specific field permits were required as the sites are public traditional grazing areas.

• In accordance with data availability requirement, we have uploaded our raw measurement data as ’s1_Data.xlsx' to ensure the minimal data set is available for reproducibility.

• We have provided the author-generated R code used for statistical analysis and mineral intake modeling as ’s1_Code.R'.

Reviewer Comments

Reviewer #1: General Evaluation

This study provides a valuable quantitative assessment of a traditional livestock feeding practice by evaluating the mineral composition and physicochemical properties of "hora" (natural mineral water) and its surrounding mineral-enriched soils in southwestern Ethiopia. By analyzing samples from the Bedele, Dabo, Gechi, and Borecha districts, the authors effectively highlight the potential of these resources as a strategic natural source of sodium and selenium, while also correctly identifying the toxicological risks associated with excessive iron, molybdenum, and manganese in the soil.

The methodology, incorporating APHA standards for water and ICP-OES analysis for mineral quantification, is technically sound. Furthermore, modeling the potential daily mineral intake based on established water and dry matter consumption formulas for tropical cattle is a highly commendable approach that bridges analytical chemistry with practical animal nutrition. The manuscript aligns well with the publication criteria of PLOS ONE, presenting original research with rigorously supported conclusions. However, addressing a few methodological limitations and contextual factors in the discussion will significantly strengthen the final manuscript.

Author Response: We thank the reviewer for recognizing the value of this quantitative assessment and for the positive comments regarding our methodology and the bridge between analytical chemistry and animal nutrition.

Specific Comments

Baseline and Control Data: As the authors rightly acknowledged in the discussion, the study currently lacks a comparative baseline from sites distal to the hora springs. Without these control samples for background water and soil, quantifying the exact magnitude of mineral enrichment relative to the region’s basal environmental status remains difficult. I recommend expanding the discussion slightly to address how this limitation might influence the interpretation of the net dietary mineral balance.

Response: We have expanded the Methodological limitation section of the discussion. We now explicitly acknowledge that without distal control samples, we cannot precisely quantify the exact magnitude of enrichment relative to the region’s basal environmental status. This addition clarifies that our findings represent the specific characteristics of the hora sites rather than a regional comparison.

Seasonal Variability: The sampling was exclusively conducted during the dry season (December-February). It is highly probable that precipitation during the wet season would dilute the total dissolved solids (TDS) and alter specific mineral concentrations. Please add a brief paragraph discussing the potential impact of seasonal dynamics on water palatability, mineral concentration, and consequently, the variations in the cattle’s daily mineral intake throughout the year.

Author Response: We have added a new paragraph to the subsection Limitations and Future Directions at the end of discussion and addressing seasonal variations (Line 337-342, page 17). We acknowledge that dry-season sampling may present peak concentrations and discuss how rainy-season precipitation may dilute minerals and influence the cattle’s water source preference and daily mineral intake patterns.

Bioavailability vs. Gross Intake: The daily soil ingestion was reasonably estimated at 0.5 kg DM/hd/day. While the gross mineral intake calculations presented in Table 4 are helpful, the alkaline pH recorded in the soil samples (e.g., reaching up to 8.1 in Bedele and 8.28 in Dabo) does not favor the optimal absorption of most minerals in the gastrointestinal tract. Furthermore, the extremely high levels of iron, molybdenum, and manganese could induce secondary copper deficiency due to strong antagonistic interactions. I suggest explicitly emphasizing that the calculated gross intake does not equal the bioavailable intake, particularly under these alkaline and highly antagonistic matrix conditions.

Author Response: We have updated the discussion and the description of Table 4 to emphasize that values represent gross intake. We added text explaining that the recorded alkaline pH (reaching 8.28 in Dabo) likely hinders absorption and discussed the risk of secondary copper deficiency caused by antagonistic interactions involving Fe, Mo, and Mn. Furthermore, we now discuss the risk of secondary copper deficiency caused by the antagonistic interactions of the extremely high levels of Fe, Mo, and Mn identified in our soil analysis.

Methodological Clarity (HG-ICP-OES): The use of microwave digestion and subsequent hydride generation (HG-ICP-OES) for selenium determination is an excellent methodological choice to enhance sensitivity and minimize matrix interferences. To further validate this analytical rigor, please consider adding a brief statement in the methodology section regarding the recovery rates or the limits of quantification (LOQ) specific to this selenium analysis.

Author Response: We agree that analytical transparency is vital. While specific recovery rates were not indexed in the final lab report, we have updated the Materials and Methods to reflect the instrumental optimization (daily standardization and calibration using Inorganic Ventures stock solutions) and adherence to standard manufacturer protocols for trace element sensitivity.

Reviewer #2: Editor

I appreciate the trust placed in me to review the article titled: “Nutritional Value of Alfalfa and Mineral-Enriched Soil for Livestock: Evaluation of Mineral Composition and Physicochemical Properties in Southwest Ethiopia.” The article is well-structured, and the study’s objective is relevant.

Author Response: We thank the Reviewer for their appreciative comments regarding the structure and relevance of our study. We note that the Reviewer’s summary mentioned 'Alfalfa'; however, we would like to clarify that our study focus and manuscript title remain: Nutritional Value of Hora and Mineral-enriched Soil for Cattle: Evaluation of Mineral Composition and Physicochemical Properties in Southwestern Ethiopia.

Some minor grammatical details have been identified. Regarding the citation of references within the document, a careful review is necessary, and the numbering should be restructured. The inconsistencies begin at the foot of Table 1, where it appears as: CCME (2025) and should be CCME [27].

Author Response: We have restructured the numbering of references to strictly follow PLOS ONE style. Specifically, in Table 1, the citation "CCME (2025)" has been corrected to the bracketed format: CCME [27].

Other grammatical details are observed on page 5, lines 129, 130 Please review the punctuation and subscript in the chemical formula NH3, line 132.

Author Response: We have conducted a thorough review of the punctuation and formatting on page 5. Chemical notations have been corrected to include proper subscripts consistently throughout the manuscript.

Attachment Submitted filename: Response to Reviewers.docx

# Editor report: Decision Letter 1

**DOI:** 10.1371/journal.pone.0348764.r003

**Authors:** Elma Izze Magalhães

21 Apr 2026

Nutritional value of hora and mineral-enriched soil for cattle: Evaluation of mineral composition and physicochemical properties in southwestern Ethiopia.

PONE-D-25-68057R1

Dear Dr. Miresa,

We’re pleased to inform you that your manuscript has been judged scientifically suitable for publication and will be formally accepted for publication once it meets all outstanding technical requirements.

Within one week, you’ll receive an e-mail detailing the required amendments. When these have been addressed, you’ll receive a formal acceptance letter and your manuscript will be scheduled for publication.

An invoice will be generated when your article is formally accepted. Please note, if your institution has a publishing partnership with PLOS and your article meets the relevant criteria, all or part of your publication costs will be covered. Please make sure your user information is up-to-date by logging into Editorial Manager at Editorial Manager® and clicking the ‘Update My Information' link at the top of the page. For questions related to billing, please contact billing support.

If your institution or institutions have a press office, please notify them about your upcoming paper to help maximize its impact. If they’ll be preparing press materials, please inform our press team as soon as possible -- no later than 48 hours after receiving the formal acceptance. Your manuscript will remain under strict press embargo until 2 pm Eastern Time on the date of publication. For more information, please contact onepress@plos.org.

Kind regards,

Elma Izze Da Silva Magalhães

Academic Editor

PLOS One

# Editor report: Acceptance letter

**DOI:** 10.1371/journal.pone.0348764.r004

**Authors:** Elma Izze Magalhães

PONE-D-25-68057R1

PLOS One

Dear Dr. Miresa,

I'm pleased to inform you that your manuscript has been deemed suitable for publication in PLOS One. Congratulations! Your manuscript is now being handed over to our production team.

At this stage, our production department will prepare your paper for publication. This includes ensuring the following:

* All references, tables, and figures are properly cited

* All relevant supporting information is included in the manuscript submission,

* There are no issues that prevent the paper from being properly typeset

You will receive further instructions from the production team, including instructions on how to review your proof when it is ready. Please keep in mind that we are working through a large volume of accepted articles, so please give us a few days to review your paper and let you know the next and final steps.

Lastly, if your institution or institutions have a press office, please let them know about your upcoming paper now to help maximize its impact. If they'll be preparing press materials, please inform our press team within the next 48 hours. Your manuscript will remain under strict press embargo until 2 pm Eastern Time on the date of publication. For more information, please contact onepress@plos.org.

You will receive an invoice from PLOS for your publication fee after your manuscript has reached the completed accept phase. If you receive an email requesting payment before acceptance or for any other service, this may be a phishing scheme. Learn how to identify phishing emails and protect your accounts at https://explore.plos.org/phishing.

If we can help with anything else, please email us at customercare@plos.org.

Thank you for submitting your work to PLOS ONE and supporting open access.

Kind regards,

PLOS ONE Editorial Office Staff

on behalf of

Dr. Elma Izze Da Silva Magalhães

Academic Editor

PLOS One
